# INTEREST GROUP SESSION—KOREAN AND KOREAN AMERICAN AND AGING: UNDERSTANDING THE HEALTH AND WELL-BEING OF OLDER KOREAN AMERICANS FROM CULTURAL PERSPECTIVES

**DOI:** 10.1093/geroni/igz038.1509

**Published:** 2019-11-08

**Authors:** Miyong T Kim, David Chiriboga, Barbara Yee

**Affiliations:** 1 University of Texas at Austin, Austin, Texas, United States; 2 University of South Florida, Tampa, Florida, United States; 3 University of Hawaii at Mānoa, Honolulu, Hawaii, United States

## Abstract

Despite tremendous progress in improving health in the U.S. in recent years, many ethnic minority populations still experience significant health disparity gaps that stem from a lack of valid research and culturally sensitive service infrastructures for those populations. Our previous research indicates that overwhelming numbers of older Korean Americans, a first-generation immigrant group, suffer not only from health issues but also from a lack of self-confidence and a feeling of social isolation because of language and cultural barriers. In addition, many older Korean Americans lack personal resources and health literacy to overcome those barriers when they attempt to access the complicated U.S. health care system. The purpose of this symposium is to promote the understanding of the health and well-being of older Korean Americans and identify their vulnerabilities and resilience. A series of five community-based studies of older Korean Americans conducted in multiple locations (e.g., FL, HI, TX, NY, CA, MD, VA, DC, MN, and IL) that covers diverse topics on health and well-being (e.g., health literacy, chronic disease management intervention, physical/mental/oral/cognitive health, end-of-life issues, diabetes, cancer, and dementia), using various methodologies (e.g., quantitative surveys, qualitative interviews, focus groups, and randomized controlled trials) will be presented. The issues of diversities and disparities will be discussed from the cultural perspectives, as well as implications for future research and practice.

